# Natrium Benzoate Alleviates Neuronal Apoptosis via the DJ-1-Related Anti-oxidative Stress Pathway Involving Akt Phosphorylation in a Rat Model of Traumatic Spinal Cord Injury

**DOI:** 10.3389/fnmol.2019.00042

**Published:** 2019-02-22

**Authors:** Liansheng Gao, Zhongyuan Zhang, Weilin Xu, Tao Li, Guangyu Ying, Bing Qin, Jianru Li, Jingwei Zheng, Tengfei Zhao, Feng Yan, Yongjian Zhu, Gao Chen

**Affiliations:** ^1^Department of Neurosurgery, Second Affiliated Hospital, School of Medicine, Zhejiang University, Hangzhou, China; ^2^Department of Orthopedics, Second Affiliated Hospital, School of Medicine, Zhejiang University, Hangzhou, China

**Keywords:** natrium benzoate, DJ-1, oxidative stress, reactive oxygen species, apoptosis, traumatic spinal cord injury

## Abstract

This study aimed to explore the neuroprotective effects and mechanisms of natrium benzoate (NaB) and DJ-1 in attenuating reactive oxygen species (ROS)-induced neuronal apoptosis in traumatic spinal cord injury (t-SCI) in rats. T-SCI was induced by clip compression. The protein expression and neuronal apoptosis was evaluated by Western blotting, double immunofluorescence staining and transmission electron microscope (TEM). ROS level, spinal cord water content (SCWC) and Evans blue (EB) extravasation was also examined. Locomotor function was evaluated by Basso, Beattie, and Bresnahan (BBB) and inclined plane test (IPT) scores. We found that DJ-1 is expressed in spinal cord neurons and increased after t-SCI. At 24 h post-injury, the levels of DJ-1, p-Akt, SOD2, ROS, p-p38 MAPK/p38 MAPK ratio, and CC-3 increased, while the Bcl-2/Bax ratio decreased. NaB upregulated DJ-1, p-Akt, and SOD2, decreased ROS, p-p38 MAPK/p38 MAPK ratio, and CC-3, and increased the Bcl-2/Bax ratio, which were reversed by DJ-1 siRNA. The proportion of CC-3- and TUNEL-positive neurons also increased after t-SCI and was reduced by NaB. These effects were reversed by MK2206. Moreover, the level of oxDJ-1 increased after t-SCI, which was decreased by DJ-1 siRNA, NaB or the combination of them. NaB also reduced mitochondrial vacuolization, SCWC and EB extravasation, and improved locomotor function assessed by the BBB and IPT scores. In conclusion, NaB increased DJ-1, and thus reduced ROS and ROS-induced neuronal apoptosis by promoting Akt phosphorylation in t-SCI rats. NaB shows potential as a therapeutic agent for t-SCI, with DJ-1 as its main target.

## Introduction

Traumatic spinal cord injury is one of the most serious injuries among all traumas worldwide and generally results in severe and permanent neurological dysfunction and neurodegeneration (Seo et al., 2015). The pathophysiology of t-SCI involves a primary injury followed by secondary damage. Following trauma, the primary injury occurs immediately, which comprises of a tissue contusion, axonal fracture, and a vascular rupture. The secondary injury results from diverse molecular, cellular, and biochemical responses induced by the primary injury and may persist for hours, days, or weeks; these responses include the oxidative stress reaction (Lam et al., 2013; Fatima et al., 2015).

During oxidative stress, molecular oxygen is inadequately reduced in the mitochondria, resulting in excessive levels of ROS. Under normal conditions, a balance between ROS generation and degradation is maintained (Droge, 2002; Valko et al., 2007). However, the ROS level is significantly increased in the traumatic spinal cord (Ahuja et al., 2017). Excessive ROS leads to various types of destructive effects such as lipid peroxidation, protein oxidation, and DNA damage. Additionally, ROS can activate specific cascades that induce cell death (Sugawara and Chan, 2003). It was reported that ROS can induce cell apoptosis by activating the p38 MAPK signaling pathway (Wang et al., 2003; Huang et al., 2018), which plays an important role in the pathogenesis of t-SCI. Neuronal apoptosis is one of the many factors contributing to the poor prognosis of t-SCI (Niu and Yip, 2011). Thus, reducing oxidative stress-induced neuronal apoptosis following t-SCI, may have important therapeutic effects.

Natrium benzoate, the sodium salt of an aromatic carboxylic acid, is a metabolite of cinnamon. It is commonly used as a flavoring material and as a preservative for a variety of foods and cosmetics (Nair, 2001) and has a long history of medical use. Williams and Lock (2005) reported that NaB attenuated D-serine-induced nephrotoxicity in rats. It was previously demonstrated that treatment with NaB upregulated T_reg_ cells and ameliorated relapsing-remitting experimental allergic encephalomyelitis in MS mice (Brahmachari and Pahan, 2007). NaB was also used as a drug to treat hyperammonemia caused by hepatic metabolic defects, such as urea cycle defects in children (approved by the US FDA) (Scaglia et al., 2004; Gropman et al., 2007; Misel et al., 2013). In the CNS, NaB can inhibit activated glial cells to express a variety of proinflammatory factors (Brahmachari et al., 2009). Modi et al. (2015) found that NaB decreased the generation of ROS in activated microglia. Jana et al. (2013) reported that NaB increased the expression of neurotrophic factors (BDNF and NT-3) via the PKA-CREB pathway both *in vivo* and *in vitro*. Recently, an increasing number of studies have demonstrated that NaB has neuroprotective effects in AD, PD, and other neurodegenerative disorders by upregulating the expression of DJ-1 (Khasnavis and Pahan, 2012, 2014). However, the potential mechanisms remain unclear.

DJ-1, also known as PARK7, is a highly conserved protein expressed in the tissues of nearly all organisms ranging from bacteria to humans (Bonifati et al., 2003). The DJ-1 gene was originally identified as an oncogene and mutations in this gene were found to be responsible for familial PD (Nagakubo et al., 1997). In the CNS, DJ-1 is expressed in neurons, astrocytes, and microglia (Bandopadhyay et al., 2004; Kim et al., 2013). DJ-1 mainly localizes in the cytoplasm and a small amount is found in the nucleus and mitochondria. The correlation between its subcellular distribution and biological function remains unclear (Junn et al., 2009). DJ-1 has diverse functions and is involved in multiple physiological activities, such as oncogenesis (Nagakubo et al., 1997), protein-RNA interactions (Hod et al., 1999; Van Der Brug et al., 2008; Blackinton et al., 2009a), transcriptional regulation (Xu et al., 2005; Zhong et al., 2006; Takahashi-Niki et al., 2017), molecule chaperone (Shendelman et al., 2004; Zhou et al., 2006; Batelli et al., 2008), fertilization (Okada et al., 2002; Yoshida et al., 2003), mitochondrial function regulation (Shimizu et al., 2016), glycation damage prevention (Advedissian et al., 2016), and, most importantly, the oxidative stress reaction (Pantcheva et al., 2014). DJ-1 has shown neuroprotective effects in neurodegenerative diseases and ischemic stroke. Injection of DJ-1 into the substantia nigra reduced neuronal death and improved motor functions in a rat model of PD (Inden et al., 2006). DJ-1 protected against ischemia and reperfusion damage in focal cerebral ischemia rats (Yanagisawa et al., 2008; Yanagida et al., 2009). The loss of DJ-1 aggravated neuronal impairment caused by cerebral ischemia (Aleyasin et al., 2007).

The potential neuroprotective mechanism of DJ-1 may rely on its ability to rescue cells from oxidative stress (Pantcheva et al., 2014). Several studies have clarified the important functions of DJ-1, including oxidative stress sensing and ROS scavenging in the brain (Taira et al., 2004; Zhang et al., 2005). It has been reported that DJ-1 is sensitive to oxidation and its protein level is upregulated by oxidative stress induction (Mitsumoto et al., 2001; Jin et al., 2005). DJ-1 knockdown cells or DJ-1 knockout mice were prone to suffering cell death after treatment with H_2_O_2_ or other neurotoxins (Yokota et al., 2003; Martinat et al., 2004; Chen et al., 2005; Kim et al., 2005b). The exact mechanisms of how DJ-1 protects against oxidative stress remain unclear, but it may enhance the phosphorylation of Akt (Kim et al., 2005a; Yang et al., 2005; Di Segni et al., 2006; Gorner et al., 2007; Van Der Brug et al., 2008; Aleyasin et al., 2010; Wang et al., 2014; Zhang et al., 2016). However, whether this capacity of DJ-1 functions in t-SCI is unknown.

DJ-1 is a potential target for treating various neurodegenerative diseases and ischemic stroke. However, the effects of DJ-1 on t-SCI and its possible mechanisms have not been investigated. In the current study, we confirmed that NaB treatment reduces oxidative stress-induced neuronal apoptosis, thus promoting functional recovery after t-SCI in rats, possibly via DJ-1-mediated Akt phosphorylation.

## Materials and Methods

### Animal Model

Adult male SD rats (250–300 g) were obtained from the Slac Laboratory Animal Co., Ltd. (Shanghai, China). All rats were reared in an environment of constant temperature and humidity and with a normal circadian rhythm. The rats were intraperitoneally injected with 1% pentobarbital (40 mg/kg) before surgery. A 2-cm midline incision was made at the level of the T10 vertebra. After exposure of the lamina, laminectomy of T10 was performed to reveal the spinal cord. Next, a vascular clip (30 g force, INS 14120, Kent Scientific, Torrington, CT, United States) was used to clamp the spinal cord for 30 s without destroying the dura mater, which would cause a spinal cord compression injury (Liang et al., 2010). The sham rats were subjected to a similar surgery but without the clamp. Each rat was intraperitoneally injected with 5 mL saline for a water complement after surgery and was kept warm during recovery. Bladder massages were performed twice daily to facilitate urination until the recovery of urinary function.

### Drugs and Small Interfering RNA (siRNA)

Natrium benzoate (100 mg/kg, Sigma-Aldrich, St. Louis, MO, United States) was dissolved in 100 μL of water and the rats were treated with NaB-containing water via gavage at 1 h after t-SCI (Khasnavis and Pahan, 2014; Kundu et al., 2016). An Akt inhibitor, MK2206, (100 μg, Selleck Chemicals, Houston, TX, United States) was dissolved in dimethyl sulfoxide and further diluted in 10 μL of sterile saline. The rats were treated with MK2206-containing normal saline via intrathecal injections at 1 h after t-SCI (Yan et al., 2017).

Two target-specific siRNAs disturbing rat DJ-1 mRNA mixtures (sense: 5’-CCCAUUGGCUAAGGACAAATT-3’, 5’-UGGAGACGGUCAUCCCUGUTT-3’) or scramble siRNA (sense: 5’-UUCUCCGAACGUGUCACGUTT-3’) obtained from Thermo Fisher Scientific (Waltham, MA, United States) were dissolved in Entranster^TM^-*in vivo* transfection reagent (500 pmol/10 μL, Engreen Biosystem, Beijing, China). The rats were intrathecally injected with siRNA solution at 48 h before t-SCI as previously described (Figueroa et al., 2016).

### Intrathecal (i.t.) Injection

Intrathecal injections were administered as previously described (Hylden and Wilcox, 1980). Briefly, the rat was fixed in one hand with its back arched, while the other hand held a syringe positioned at 20° over the spine with its needle tip pointing forward to puncture the subarachnoid space via the intervertebral space between L5 and L6. The injection speed was 2 μL/min. After injection, the needle was kept *in situ* for an additional 10 min before seceding. The sham rats were subjected to the same puncture but without drug injection.

### Study Design

#### Experiment 1

We randomly allocated the rats into seven groups: sham (*N* = 12), t-SCI 3 h (*N* = 6), t-SCI 6 h (*N* = 6), t-SCI 12 h (*N* = 6), t-SCI 24 h (*N* = 12), t-SCI 48h (*N* = 6), and t-SCI 72 h (*N* = 6). Six rats in each group were used to detect the changes in DJ-1 and p-Akt expression over time by Western blotting. Six rats in the sham and t-SCI 24 h groups were used for double IF staining of DJ-1 and NeuN.

#### Experiment 2

To investigate the functions of DJ-1, we randomly distributed the rats into six groups: sham (*N* = 24), t-SCI + vehicle (*N* = 24), t-SCI + scramble siRNA (*N* = 6); t-SCI + DJ-1 siRNA (*N* = 6), t-SCI + NaB (*N* = 24), and t-SCI + NaB + DJ-1 siRNA (*N* = 6). At 24 h post-injury, six rats from each group were used to quantify the levels of DJ-1, oxDJ-1, Akt, SOD2, p38 MAPK, Bcl-2, Bax, and CC-3 by Western blotting. ROS were measured in the other six rats in these groups. EB extravasation and SCWC were detected using the other six rats in the sham, t-SCI + vehicle, and t-SCI + NaB groups, respectively. Another six rats in these groups were used to observe the ultrastructure of the cells by TEM.

#### Experiment 3

To examine the long-term functions of DJ-1 in neurological improvement, we randomly allotted the rats into three groups: sham (*N* = 6), t-SCI + vehicle (*N* = 6), and t-SCI + NaB (*N* = 6). All rats were treated for seven consecutive days post-injury. The BBB and IPT scores were determined before and at 1, 3, 7, 14, 21, and 28 days after treatment in all groups.

#### Experiment 4

To analyze the mechanism of action of DJ-1, we randomly assigned the rats into five groups: sham (*N* = 6), t-SCI + vehicle (*N* = 18), t-SCI + NaB (*N* = 18), t-SCI + MK2206 (*N* = 18), and t-SCI + NaB + MK2206 (*N* = 18). At 24 h post-injury, six rats in each group, except the sham group, were used to quantify the expression levels of DJ-1, Akt, SOD2, p38 MAPK, Bcl-2, Bax, and CC-3 by Western blotting. ROS levels were measured in the other six rats in these groups. Another six rats in each group were used for TUNEL, CC-3, and NeuN double IF staining.

**FIGURE 1:**
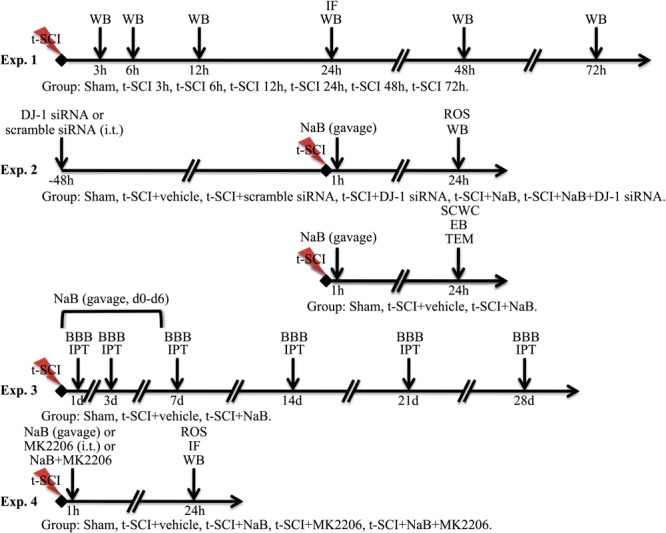
Detailed experimental design.

The detailed experimental design is shown in Figure 1.

### Motor Function Assessment

The locomotor functions of rats in each group were evaluated by determining the BBB (Basso et al., 1995) and IPT (Rivlin and Tator, 1977) scores on days 1, 3, 7, 14, 21, and 28 after t-SCI. The detailed BBB scores are shown in Table 1. IPT was conducted as follows. The long axis of the rat body and long axis of the inclined plate were placed vertically. Gradually, the angle between the inclined plate and horizontal plane was increased until the rats could just remain on the inclined plate for 5 s, and this angle was recorded. The researcher was blinded to the rat group assignments during evaluation. Each rat was assessed three times and the average value was taken as the final score.

**Table 1:** BBB score.

Score	Description
0	No hind limb movement.
1	Slight movement of one or two joints, typically hip and/or knee joints.
2	One joint moves extensively, or one joint moves extensively and another joint moves slightly.
3	Extensive movement of both joints.
4	All three joints of the hind limb can move slightly.
5	Two joints move slightly, and the third can move extensively.
6	Two joints move extensively, and the third joint can move slightly.
7	All three joints of the hind limb can move extensively.
8	The palm can touch the ground under non-load-bearing conditions.
9	Occasionally, the palm can support or back can move under load-bearing conditions, no movement under palm support.
10	Occasionally, the palm moves under load-bearing conditions, and there is no coordinated movement of fore-hind limbs.
11	More palm load-bearing movement, but no coordinated movement of fore-hind limbs.
12	More palm load-bearing movement, occasional coordinated movement of fore-hind limbs.
13	Common palm load-bearing movement, common coordinated movement of fore-hind limb.
14	Persistent palm load-bearing movement and coordinated movement of fore-hind limb; or common palm movement and persistent coordinated movement of fore-hind limb, occasional back movement.
15	Persistent palm movement and coordinated movement of fore-hind limbs, no or occasional grip during fore limb advancement; the position of active claw is parallel to the body at initial contact.
16	Persistent palm movement and coordinated movement of fore-hind limbs, common grip during fore limb advancement; the position of active claw is parallel to the body at initial contact and rotated after weight transfer.
17	Persistent palm movement and coordinated movement of fore-hind limbs, common grip during fore limb advancement; the position of active claw is parallel to the body at initial contact and after weight transfer.
18	Persistent palm movement and coordinated movement of fore-hind limbs, persistent grip during fore limb advancement; the position of active claw is parallel to the body at initial contact and rotated after weight transfer.
19	Persistent palm movement and coordinated movement of fore-hind limbs, persistent grip during fore limb advancement; the position of active claw is parallel to the body at initial contact and after weight transfer. The tail sometimes or always droops.
20	Persistent palm movement, coordinated gait, toe grip, the position of active claw is parallel to the body at initial contact and after weight transfer. The trunk is unstable, and the tail keeps tilting.
21	Persistent palm movement, coordinated gait, toe grip, the position of active claw is always parallel to the body, the trunk is stable, and the tail keeps tilting.

### SCWC Examination

The SCWC was detected using the wet–dry method (Hu et al., 2015). Spinal cord samples of 1.5-cm long, where the injured site was centered, were obtained. The samples were weighted immediately (wet weight). Next, they were dried at 95°C for 72 h and then weighted (dry weight). The researcher was blinded to the rat group assignments. The results were presented as the water content (g) in unit wet tissue (g).

### Blood-Spinal Cord Barrier (BSCB)Leakage

Blood-spinal cord barrier leakage was assessed by EB extravasation (Li et al., 2014; Zhou et al., 2017). EB solution (3%, 45 mg/kg, Sigma-Aldrich) was administrated through the femoral vein after anesthetization. One hour later, spinal cord samples of 1.0-cm long, where the injured site was centered, were obtained after transcardial perfusion with 0.1M PBS. The sample was homogenized in 3 mL 50% trichloroacetic acid and centrifuged at 12,000 × *g* at 4°C for 20 min. Next, 1 mL of the supernatant was mixed with 1 mL of ethanol and trichloroacetic acid solution (1:3) and incubated at 4°C overnight. The sample was then centrifuged at 15,000 × *g* at 4°C for 30 min. The supernatant was examined at an emission wavelength of 680 nm and excitation wavelength of 620 nm with a microplate reader (Bio-Tek Instruments, Winooski, VT, United States). The fluorescence value was converted to an EB content (μg) according to the standard curve. The researcher was blinded to the group assignments of the rats. Each test was conducted twice, and the average value was taken as the result. The results were expressed as the EB content (μg) in unit tissue (g).

### Western Blot Analysis

Spinal cord samples of 0.5-cm long, where the injured site was centered, were obtained after transcardial perfusion with 0.1M PBS. The tissues were homogenized in RIPA lysis buffer containing protease inhibitors (1%) and phosphates inhibitors (1%), and then centrifuged at 12,000 × *g* at 4°C for 15 min. The protein concentration of the supernatant was measured using a BCA protein assay kit (Pierce, Rockford, IL, United States). Each sample was diluted to the same protein concentration (4 μg/μL) with RIPA and resuspended in loading buffer. An equal volume of protein samples (10 μL) was loaded into each well of a 12% SDS-PAGE gel. The proteins were separated by electrophoresis and then transferred onto a polyvinylidene fluoride membrane (Bio-Rad Laboratories, Hercules, CA, United States). The membrane was blocked in 10% skim milk for 1 h. After rinsing with Tris-buffered saline and Tween 20, specific primary antibodies including anti-DJ-1 (1:20,000, ab76008, Abcam, Cambridge, United Kingdom), anti-oxDJ-1 (1:5000, ab169520, Abcam), anti-p-Akt (1:2000, CST#4060s, Cell Signaling Technology, Danvers, MA, United States), anti-Akt (1:1000, CST#9272s, Cell Signaling Technology), anti-SOD2 (1:5000, ab13533, Abcam), anti-p-p38 MAPK (1:1000, ab4822, Abcam), anti-p38 MAPK (1:1000, ab31828, Abcam), anti-Bax (1:1000, ab32503, Abcam), anti-Bcl-2 (1:500, ab59348, Abcam), anti-CC-3 (1:500, ab13847, Abcam), and anti-β-actin (1:5000, ab8226, Abcam) were incubated with the membranes at 4°C overnight. After rinsing, the membranes were treated with appropriate secondary antibodies (1:10,000, ZB-2301 or ZB-2305, Zhongshan Golden Bridge, Beijing, China) for 1 h at 25°C and rinsed again. The membranes were immersed in a mixture of equal amounts of the A and B solutions from an ECL kit (Immobilon, Millipore, Billerica, MA, United States) and then observed using a chemical imager (Bio-Rad Laboratories) to detect the proteins.

The gray level of each band was analyzed using ImageJ software (National Institutes of Health, Bethesda, MD, United States). The results were displayed as the ratios of gray levels of the protein of interest to the internal control proteins and normalized against the sham group.

### IF and TUNEL Dyeing

Spinal cord samples of 1.0-cm long, where the injured site was centered, were obtained after transcardial perfusion with 0.1M PBS. Following fixation in 4% paraformaldehyde at 4°C for 24 h and dehydration in 30% sucrose solution at 4°C for 72 h, three axial frozen sections (20 μm) were obtained from the proximal spinal cord, while another three sections were obtained from the distal spinal cord 2 mm away from the center of the injury site, respectively, giving a total of six sections from each sample (Kjell et al., 2016; Wang et al., 2017). After disrupting the cell membranes with 0.3% Triton X-100 for 15 min, blocking with 10% donkey serum for 2 h at 25°C, and rinsing with 0.01M PBS, specific primary antibodies including anti-DJ-1 (1:500, ab76008, Abcam), anti-CC-3 (1:200, ab13847, Abcam), and anti-NeuN (1:500, ab104224, Abcam) were incubated with the sections at 4°C overnight. The sections were incubated for 2 h at 25°C with appropriate secondary antibodies (1:500, Invitrogen, Thermo Fisher Scientific) or TUNEL dye liquor (Roche, Basel, Switzerland). DAPI (1 μg/mL, Sigma-Aldrich) was used to stain the nucleus, and then the sections were mounted. Finally, the sections were observed with a fluorescence microscope (Olympus, Tokyo, Japan) and the photos were processed with Photoshop 13.0 software (Adobe Systems, Inc., San Jose, CA, United States).

One random grey matter field per section was used to count the number of cells in the photos at 200× magnification. The expression of DJ-1 was evaluated as the mean ratio of DJ-1-positive neurons to total neurons in each group. Neuronal apoptosis was evaluated as the mean ratio of CC-3-/TUNEL-positive neurons to total neurons in each group.

### Measurement of ROS Levels

The ROS levels in each group were tested with an ROS assay kit (Jiancheng, Nanjing, China) according to the manufacturer’s instructions. Spinal cord samples of 0.5-cm long, where the injured site was centered, were obtained after transcardial perfusion with 0.1M PBS. After homogenization in 0.01M PBS and centrifugation at 1,000 × *g* at 4°C for 10 min, the supernatant from each sample (190 μL) and DCFH-DA (10 μL, 1 mol/L) were mixed in a 96-well plate at 37°C for 30 min and then examined at an emission wavelength of 525 nm and excitation wavelength of 500 nm with a microplate reader (Bio-Tek Instruments, Inc.). Additionally, the protein concentration of the supernatant was tested using a BCA protein assay kit (Pierce). The researcher was blinded to the assignments of the rats in each group. Each test was conducted twice, and the average value was taken as the result. The ROS levels were displayed as fluorescence/mg protein.

### TEM

Spinal cord samples of 0.5-cm long, where the injured site was centered, were obtained after transcardial perfusion with 0.1M PBS. Two 1-mm^3^ tissue blocks were acquired from the gray matter regions of the proximal and distal spinal cord 2 mm away from the center of the injury site, respectively, and then incubated in 2.5% glutaraldehyde for fixation overnight. The tissue blocks were subjected to rinsing, fixation in 1% osmic acid for 1 h, gradient alcohol dehydration, and embedment in araldite at 60°C overnight. Three 100-nm ultra-thin sections were obtained from each block, giving a total of six sections from each sample. A TEM (Philips Tecnai 10, Amsterdam, Netherlands) was applied to observe the slices, which had been stained with uranyl acetate and lead citrate.

Ten random neurons per section were used to count the mitochondria around the neuron nucleus in the photos at 4,200× magnification. The mitochondria vacuolization rate referred to the mean ratio of vacuolated to total mitochondria in each group.

### Statistical Analysis

Data is expressed as the mean ± SD and analyzed by *t*-test, one-way ANOVA, or two-way ANOVA and Bonferroni’s *post hoc* multiple comparisons test, with *p* < 0.05 suggesting statistical significance. The statistical analyses were conducted by GraphPad Prism for Windows (GraphPad, Inc., San Diego, CA, United States).

## Results

### Animals

**FIGURE 2:**
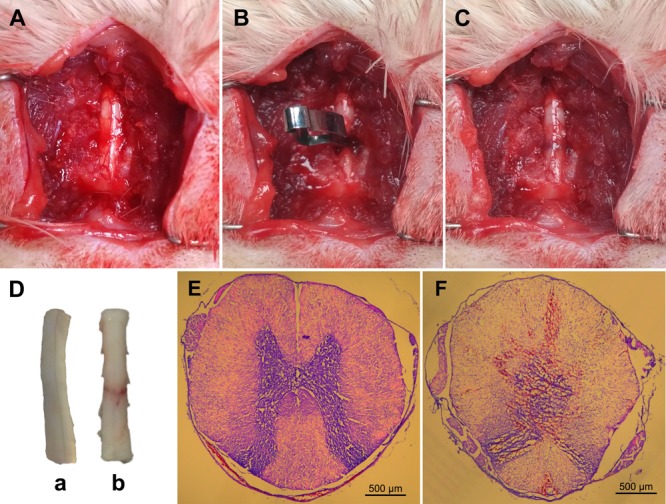
Representative figures showing the surgical procedures used to establish the clip compressive model of t-SCI: the exposed rat spinal cord **(A)**; compression of the exposed spinal cord with a vascular clip **(B)**; crushed site **(C)**. Representative spinal cord samples showing normal spinal cord (**D**, a) and crushed injury of spinal cord by vascular clip (**D**, b). Typical axial spinal cord sections from the spinal cord: normal spinal cord structure in the sham group **(E)**; spinal cord swelling, disorganization, and bleeding in the t-SCI 24 h group **(F)**.

Major physiological data were maintained within normal ranges during the surgery and no significant differences were observed among groups (Table 2). The cardinal surgical procedures used to induce spinal cord compression injury are shown in Figure 2A–C. Representative spinal cord specimens and slices in the sham and t-SCI 24 h groups are shown in Figure 2D–F.

**Table 2:** Main physiological data for rats in each group.

	HR (per min)	MAP (mmHg)	Arterial PH	pO_2_	pCO_2_
Sham	98 ± 2	103 ± 3	7.39 ± 0.02	75.5 ± 1.1	44.3 ± 0.5
t-SCI (3, 6, 12, 24, 48, and 72 h)	101 ± 5	94 ± 6	7.38 ± 0.03	76.2 ± 0.9	49.6 ± 2.1
t-SCI+vehicle	105 ± 7	103 ± 5	7.38 ± 0.02	75.5 ± 0.8	45.3 ± 2.6
t-SCI+scramble siRNA	105 ± 3	105 ± 4	7.41 ± 0.02	74.5 ± 0.8	46.2 ± 1.1
t-SCI+DJ-1 siRNA	102 ± 5	97 ± 5	7.37 ± 0.03	75.9 ± 1.2	48.6 ± 0.9
t-SCI+NaB	99 ± 8	101 ± 3	7.37 ± 0.02	73.7 ± 2.2	45.9 ± 2.9
t-SCI+NaB+DJ-1 siRNA	101 ± 5	99 ± 6	7.4 ± 0.01	75.8 ± 1.8	45.2 ± 2.3
t-SCI+MK2206	98 ± 9	106 ± 3	7.38 ± 0.01	74.7 ± 0.7	47.9 ± 1.5
t-SCI+NaB+MK2206	106 ± 3	100 ± 6	7.41 ± 0.02	75.8 ± 1.6	46.3 ± 1.2

*siRNA, small interfering RNA; NaB, natrium benzoate; t-SCI, traumatic spinal cord injury; HR, heart rate; MAP, mean arterial pressure.*

### Protein Levels of DJ-1 and p-Akt

**FIGURE 3:**
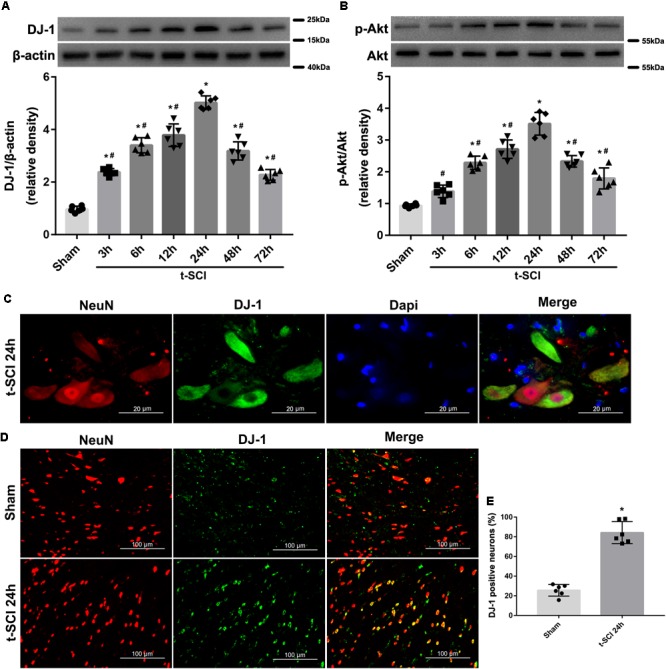
Representative Western blots showing the time course of changes in the levels of DJ-1 and p-Akt. The expression level of DJ-1 was significantly elevated at 3 h and peaked at 24 h after t-SCI, and then significantly decreased after 24 h post-injury **(A)**; the expression level of p-Akt was significantly increased at 6 h and peaked at 24 h after t-SCI, and then significantly decreased after 24 h post-injury **(B)**. *N* = 6 for each group. Data is expressed as mean ± SD and analyzed by one-way ANOVA and Bonferroni’s *post hoc* multiple comparisons test. Representative IF staining micrographs showing the proportion of DJ-1-positive neurons in each group at 24 h post-injury. DJ-1 was expressed mainly in neurons **(C)** and proportion of DJ-1-positive neurons was increased after t-SCI **(D,E)**. *N* = 6 for each group. Data is expressed as mean ± SD and analyzed by *t*-test. ^∗^*p* < 0.05 versus sham; ^#^*p* < 0.05 versus t-SCI 24 h.

Western blotting indicated that the protein level of DJ-1 began to significantly increase at 3 h and peaked at 24 h post-injury, when it was compared with the sham group (*p* < 0.05). The protein level of p-Akt began to significantly increase at 6 h and peaked at 24 h post-injury, when it was compared with the sham group (*p* < 0.05). The protein levels of both DJ-1 and p-Akt significantly decreased after 24 h post-injury but were still higher than those in the sham group (*p* < 0.05, Figure 3A,B).

### Distribution of DJ-1 in Cells

Double IF staining indicated that DJ-1 was expressed in neurons (Figure 3C). The proportion of DJ-1-positive neurons was increased at 24 h post-injury, when it was compared with the sham group (*p* < 0.05, Figure 3D,E).

### Downregulation of DJ-1 Increases Neuronal Apoptosis

**FIGURE 4:**
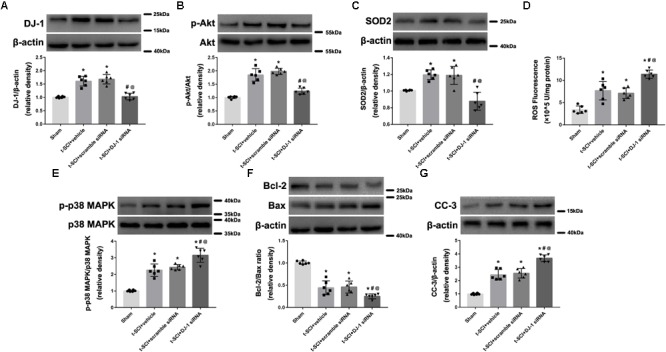
Representative Western blots showing the protein levels in each group at 24 h post-injury. The expression levels of DJ-1, p-Akt, and SOD2 were significantly increased after t-SCI, treatment with DJ-1 siRNA significantly decreased the levels of DJ-1, p-Akt, and SOD2 **(A–C)**. The ROS level, p-p38 MAPK/p38 MAPK ratio, and CC-3 level was significantly elevated, whereas the Bcl-2/Bax ratio was significantly reduced after t-SCI, and treatment with DJ-1 siRNA significantly aggravated these effects **(D–G)**. Injection of scramble siRNA did not change the expression levels of DJ-1 and the downstream targets **(A–G)**. *N* = 6 for each group. Data is expressed as mean ± SD and analyzed by one-way ANOVA and Bonferroni’s *post hoc* multiple comparisons test. ^∗^*p* < 0.05 versus sham; ^#^*p* < 0.05 versus t-SCI + vehicle; ^@^*p* < 0.05 versus t-SCI + scramble siRNA.

At 24 h post-injury, Western blotting indicated that the protein levels of DJ-1, p-Akt, and SOD2 were significantly elevated in the t-SCI + vehicle group, compared to the sham group (*p* < 0.05). Treatment with DJ-1 siRNA significantly reduced the levels of DJ-1, p-Akt, and SOD2, compared to the t-SCI + vehicle group (*p* < 0.05, Figure 4A–C). The ROS level, p-p38 MAPK/p38 MAPK ratio, and CC-3 level were significantly elevated, whereas the Bcl-2/Bax ratio was significantly reduced in the t-SCI + vehicle group, compared to the sham group (*p* < 0.05). Treatment with DJ-1 siRNA significantly elevated the ROS level, p-p38 MAPK/p38 MAPK ratio, and CC-3 level and reduced the Bcl-2/Bax ratio, compared to the t-SCI + vehicle group (*p* < 0.05, Figure 4D–G). Treatment with scramble siRNA did not alter the protein levels of DJ-1 or its downstream targets compared to those in the t-SCI + vehicle group (*p* > 0.05, Figure 4A–G).

### NaB Treatment Upregulates DJ-1 Expression and Reduces Neuronal Apoptosis

**FIGURE 5:**
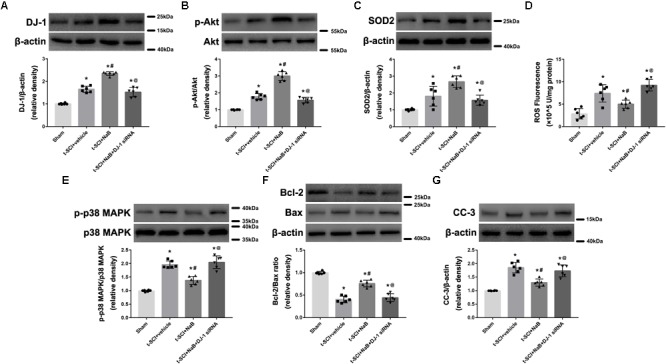
Representative Western blots showing the protein levels in each group at 24 h post-injury. Treatment with NaB significantly increased the levels of DJ-1, p-Akt, and SOD2 **(A–C)**. Treatment with NaB also significantly decreased the ROS level, p-p38 MAPK/p38 MAPK ratio, and CC-3 level and elevated the Bcl-2/Bax ratio **(D–G)**. These effects were significantly reversed by DJ-1 siRNA injection **(A–F)**. There were no differences in protein and ROS levels between the t-SCI + vehicle and t-SCI + NaB + DJ-1 siRNA groups **(A–G)**. *N* = 6 for each group. Data is expressed as mean ± SD and analyzed by one-way ANOVA and Bonferroni’s *post hoc* multiple comparisons test. ^∗^*p* < 0.05 versus sham; ^#^*p* < 0.05 versus t-SCI + vehicle; ^@^*p* < 0.05 versus t-SCI + NaB.

At 24 h post-injury, Western blotting indicated that treatment with NaB significantly elevated the protein levels of DJ-1, p-Akt, and SOD2, compared to the t-SCI + vehicle group (*p* < 0.05, Figure 5A–C). Treatment with NaB also significantly decreased the ROS level, p-p38 MAPK/p38 MAPK ratio, and CC-3 level but elevated the Bcl-2/Bax ratio, compared with the t-SCI + vehicle groups (*p* < 0.05, Figure 5D–G). These effects were obviously reversed by DJ-1 siRNA injection (*p* < 0.05 t-SCI + NaB vs. t-SCI + NaB + DJ-1 siRNA, Figure 5A–F). The protein levels displayed no obvious differences between the t-SCI + vehicle and t-SCI + NaB + DJ-1 siRNA groups (*p* > 0.05, Figure 5A–G).

### OxDJ-1 Expression Is Increased After t-SCI and Reduced by NaB Treatment

**FIGURE 6:**
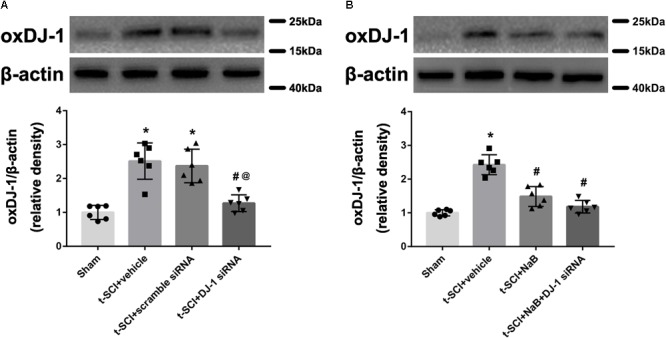
Representative Western blots showing the protein levels in each group at 24 h post-injury. The expression level of oxDJ-1 was significantly increased after t-SCI, treatment with DJ-1 siRNA significantly decreased the level of oxDJ-1, and injection of scramble siRNA did not change the expression level of oxDJ-1 **(A)**. Treatment with NaB alone or combined with DJ-1 siRNA significantly decreased the level of oxDJ-1. There were no differences in oxDJ-1 levels between the t-SCI + NaB and t-SCI + NaB + DJ-1 siRNA groups **(B)**. *N* = 6 for each group. Data is expressed as mean ± SD and analyzed by one-way ANOVA and Bonferroni’s *post hoc* multiple comparisons test. ^∗^*p* < 0.05 versus sham; ^#^*p* < 0.05 versus t-SCI + vehicle; ^@^*p* < 0.05 versus t-SCI + scramble siRNA.

At 24 h post-injury, Western blotting indicated that the protein level of oxDJ-1 was significantly elevated in the t-SCI + vehicle group, compared with the sham group (*p* < 0.05). Treatment with DJ-1 siRNA significantly reduced the level of oxDJ-1, compared with the t-SCI + vehicle group (*p* < 0.05). Treatment with scramble siRNA did not alter the protein level of oxDJ-1 compared to those in the t-SCI + vehicle group (*p* > 0.05, Figure 6A). Treatment with NaB alone or combined with DJ-1 siRNA significantly decreased the protein level of oxDJ-1, compared to the t-SCI + vehicle group (*p* < 0.05). The protein level showed no obvious differences between the t-SCI + NaB and t-SCI + NaB + DJ-1 siRNA groups (*p* > 0.05, Figure 6B).

### NaB Treatment Increases BBB and IPT Scores and Abates Spinal Cord Edema and BSCB Leakage

**FIGURE 7:**
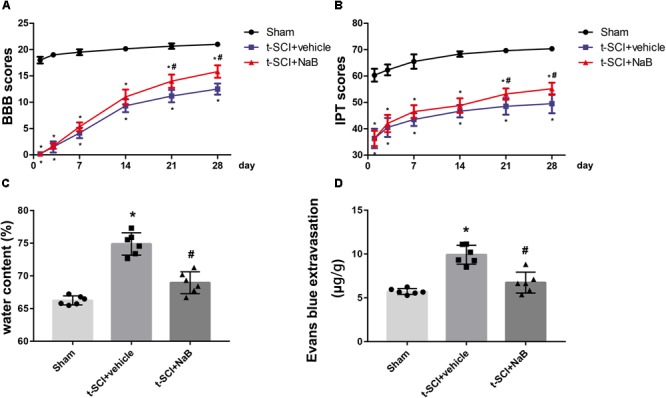
BBB and IPT scores were significantly decreased after t-SCI at each time point. Treatment with NaB significantly increased the BBB and IPT scores at days 21 and 28 post-injury **(A,B)**. *N* = 6 for each group. Data is expressed as mean ± SD and analyzed by two-way ANOVA and Bonferroni’s *post hoc* multiple comparisons test. At 24 h post-injury, treatment with NaB significantly reduced the SCWC **(C)** and EB extravasation **(D)**, which was increased after t-SCI. *N* = 6 for each group. Data is expressed as mean ± SD and analyzed by one-way ANOVA and Bonferroni’s *post hoc* multiple comparisons test. ^∗^*p* < 0.05 versus sham; ^#^*p* < 0.05 versus t-SCI + vehicle.

Rats in the sham group showed a mild decreases in their BBB and IPT scores immediately post-injury, but these values rapidly returned to normal levels. The BBB and IPT scores of each rat in the t-SCI + vehicle group and t-SCI + NaB group decreased to nearly 0 just after surgery, which continuously increased during the experimental period but were still obviously lower than those of the sham group at each time point (*p* < 0.05). NaB treatment significantly elevated the BBB and IPT scores at 21 and 28 days after t-SCI, compared to the t-SCI + vehicle group (*p* < 0.05, Figure 7A,B).

At 24 h post-injury, the SCWC and EB extravasation were significantly elevated in the t-SCI + vehicle group, compared to the sham group (*p* < 0.05). NaB treatment significantly decreased the water content and EB extravasation, compared to the t-SCI + vehicle group (*p* < 0.05, Figure 7C,D).

### Ultrastructural Changes of Spinal Cord Neurons

**FIGURE 8:**
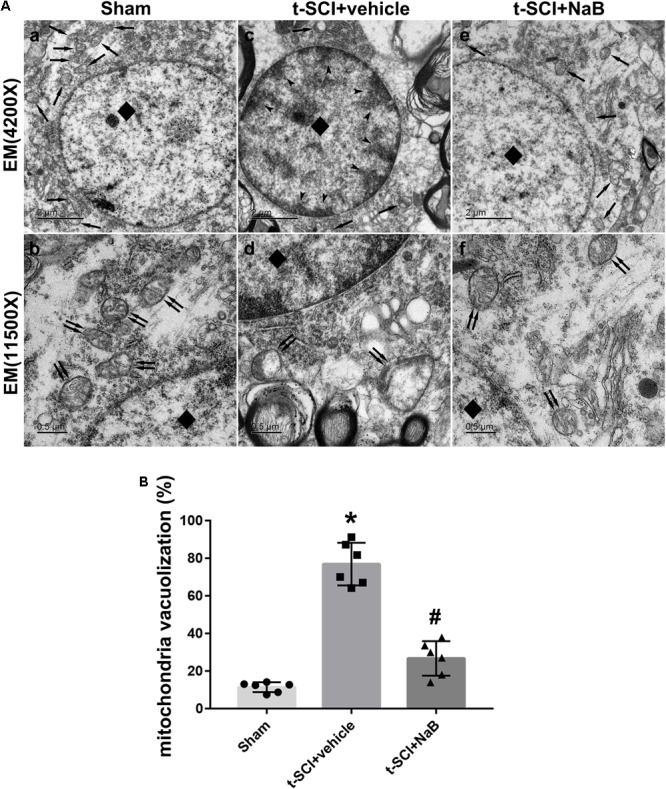
Representative TEM micrographs showing ultrastructural changes of spinal cord neurons in each group at 24 h post-injury. Normal nucleus (diamond) and mitochondrion (arrows) in the sham group **(A, a)**; normal mitochondrion (double arrows) with clear crista in the sham group **(**A, b**)**; karyopyknosis (diamond), chromatin condensation, and margination (dovetail arrows), disappearance of the nucleolus, mitochondrial loss (arrows) in the SCI + vehicle group **(**A, c**)**; mitochondrial swelling, vesiculation, and disappearance of the crista (double arrows) in the SCI + vehicle group **(**A, d**)**; treatment with NaB reversed neuronal apoptosis, and the subcellular structure reverted to near normal **(**A, e,f**)**. *N* = 6 for each group. **(B)** The proportion of mitochondrial vacuolization significantly increased after t-SCI, NaB treatment significantly decreased the proportion of mitochondrial vacuolization. Data is expressed as mean ± SD and analyzed by one-way ANOVA and Bonferroni’s *post hoc* multiple comparisons test. ^∗^*p* < 0.05 versus sham; ^#^*p* < 0.05 versus t-SCI + vehicle.

At 24 h post-injury, the spinal cord neurons in the sham group displayed plump nuclei in which the chromatin was dispersed. Moreover, the mitochondria had a normal form and distinct crista. However, in the t-SCI + vehicle group, the nuclei of the neurons were pyknosis and the chromatin showed condensation and margination. The mitochondria also swelled and vesiculated and their crista disappeared. The aberrant ultrastructure was recovered by NaB treatment (Figure 8A). The proportion of mitochondrial vacuolization was significantly increased in the t-SCI + vehicle group, compared to the sham group (*p* < 0.05), which was significantly decreased by NaB treatment, compared to the t-SCI + vehicle group (*p* < 0.05, Figure 8B).

### NaB Reduces Neuronal Apoptosis Through DJ-1/Akt-Related Anti-oxidative Stress Cascade

**FIGURE 9:**
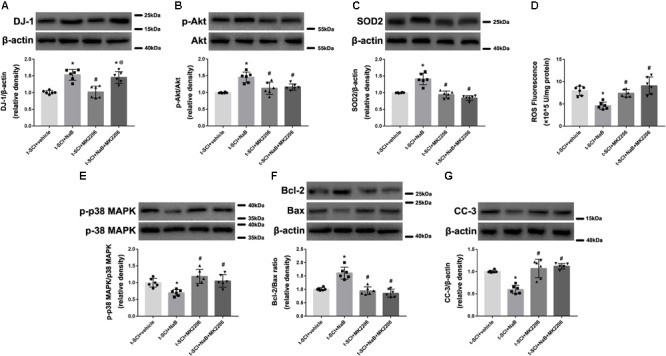
Representative Western blots showing the protein levels in each group at 24 h post-injury. Treatment with NaB significantly increased the level of DJ-1, and this upregulation was not affected by treatment with MK2206 **(A)**. The increase in the levels of p-Akt and SOD2, induced by NaB, were reversed by MK2206 **(B,C)**. The decrease in the ROS level, p-p38 MAPK/p38 MAPK ratio, and CC-3 level and the increase in the Bcl-2/Bax ratio, induced by NaB, were reversed by MK2206 **(D–G)**. Administration of MK2206 alone did not significantly alter the levels of DJ-1, its downstream proteins, and ROS **(A–G)**. *N* = 6 for each group. Data is expressed as mean ± SD and analyzed by one-way ANOVA and Bonferroni’s *post hoc* multiple comparisons test. ^∗^*p* < 0.05 versus t-SCI + vehicle; ^#^*p* < 0.05 versus t-SCI + NaB; ^@^*p* < 0.05 versus t-SCI + MK2206.

At 24 h post-injury, Western blotting indicated that NaB treatment significantly elevated the protein level of DJ-1, compared to the t-SCI + vehicle group (*p* < 0.05), and this upregulation was not altered by MK2206 treatment (Figure 9A). Treatment with NaB also significantly elevated the protein levels of p-Akt and SOD2, which reduced the level of ROS, compared to the t-SCI + vehicle group (*p* < 0.05); however, these effects were obviously reversed by MK2206 treatment (*p* < 0.05 t-SCI + NaB + MK2206 vs. t-SCI +NaB, Figure 9B–D). The p-p38 MAPK/p38 MAPK ratio and protein level of CC-3 were decreased, while the Bcl-2/Bax ratio was elevated under NaB treatment, compared to the t-SCI + vehicle group (*p* < 0.05); however, these effects were obviously reversed by MK2206 treatment (*p* < 0.05 t-SCI + NaB + MK2206 vs. t-SCI + NaB, Figure 9E–G). Moreover, administration of MK2206 alone did not obviously alter protein and ROS levels compared to those in the t-SCI + vehicle group (*p* > 0.05, Figure 9A–G).

**FIGURE 10:**
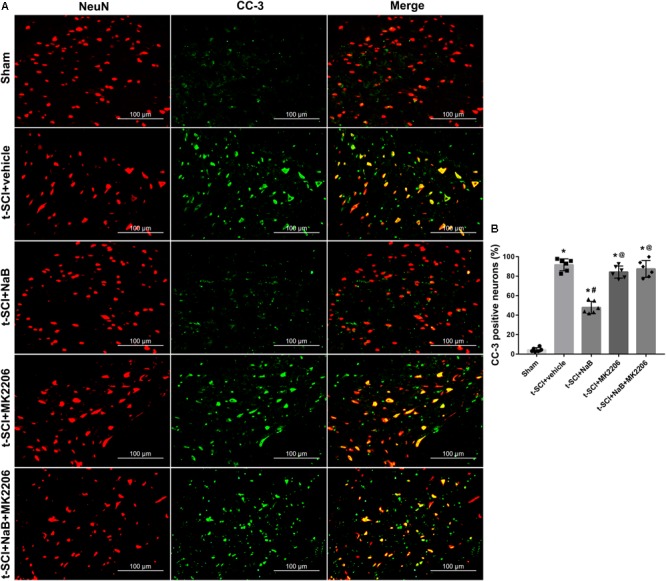
Representative IF staining micrographs showing the proportion of CC-3-positive neurons in each group at 24 h post-injury. The proportion of CC-3-positive neurons was significantly increased after t-SCI. NaB treatment significantly decreased the proportion of CC-3-positive neurons; this effect was reversed by treatment with MK2206. Administration of MK2206 alone did not significantly increase the proportion of CC-3-positive neurons **(A,B)**. *N* = 6 for each group. Data is expressed as mean ± SD and analyzed by one-way ANOVA and Bonferroni’s *post hoc* multiple comparisons test. ^∗^*p* < 0.05 versus control; ^#^*p* < 0.05 versus injury; ^@^*p* < 0.05 versus injury + NaB.

**FIGURE 11:**
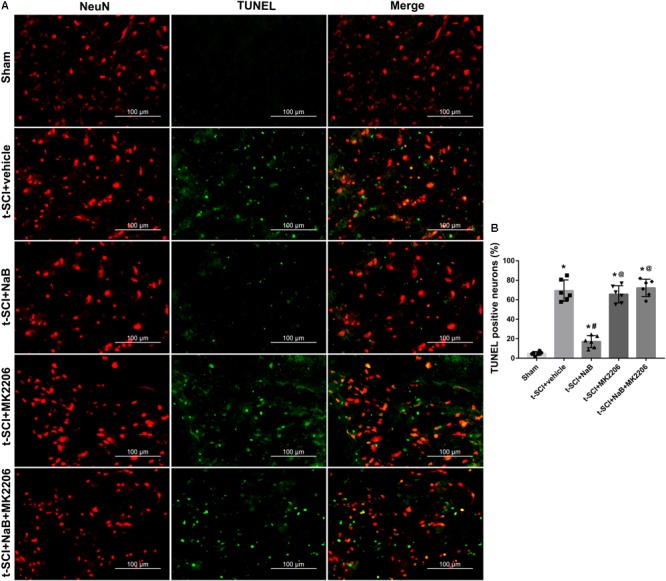
Representative IF staining micrographs showing the proportion of TUNEL-positive neurons in each group at 24 h post-injury. The proportion of TUNEL-positive neurons was significantly increased after t-SCI. NaB treatment significantly decreased the proportion of TUNEL-positive neurons; this effect was reversed by treatment with MK2206. Administration of MK2206 alone did not significantly increase the proportion of TUNEL-positive neurons **(A,B)**. *N* = 6 for each group. Data is expressed as mean ± SD and analyzed by one-way ANOVA and Bonferroni’s *post hoc* multiple comparisons test. ^∗^*p* < 0.05 versus control; ^#^*p* < 0.05 versus injury; ^@^*p* < 0.05 versus injury + NaB.

At 24 h post-injury, double IF staining indicated that t-SCI caused an increase in the proportions of CC-3- and TUNEL-positive neurons, compared to the sham group (*p* < 0.05). However, they were decreased following NaB treatment compared to those in the t-SCI + vehicle group (*p* < 0.05). These effects were obviously reversed by MK2206 treatment (*p* < 0.05 t-SCI + NaB + MK2206 vs. t-SCI + NaB). Moreover, MK2206 alone did not obviously alter the proportions of CC-3- and TUNEL-positive neurons compared to those in the t-SCI + vehicle group (*p* > 0.05, Figure 10A,B, 11A,B).

## Discussion

As a multi-functional protein, DJ-1 is involved in diverse signaling pathways, the most important being helping cells to resist oxidative stress and oxidative stress-induced apoptosis (Pantcheva et al., 2014), which is responsible for its neuroprotective functions in multiple neurological diseases. In an animal model of ischemia-reperfusion SCI, the expression of DJ-1 was increased significantly at the early stage of reperfusion (Sakurai et al., 2009). Injection of DJ-1 fusion protein protected ischemic spinal cord injury neurons by increasing the levels of antioxidant protein in cells, but the specific mechanism is unclear (Kim et al., 2014). In contrast, DJ-1 knockout makes cells vulnerable to oxidative stress (Taira et al., 2004). However, its protective effects on t-SCI and its precise molecular mechanisms remain unclear. Oxidative stress following t-SCI is a major factor causing damage to neurons. Diverse traumatic and non-traumatic SCI causes the generation of excessive ROS and dysregulation of redox homeostasis, which contribute to pathological injury to the spinal cord (Zhang et al., 2015).

Oxidative stress-induced neuronal damage is mediated by diverse mechanisms, one of which is the apoptotic cascade. ROS can lead to cell apoptosis by activating the p38 MAPK signaling pathway (Bode et al., 2012). Gomez-Lazaro et al. (2007) suggested that ROS production activated p38 MAPK and subsequently promoted the activation of the pro-apoptotic protein Bax, which can penetrate the mitochondrial membrane to cause the release of cytochrome C and activate the intrinsic apoptosis pathway in SH-SY5Y cells. Choi et al. (2004) reported that ROS initiated the p38 MAPK signaling pathway, leading to the activation of both mitochondrial and extra-mitochondrial apoptotic pathways in PD cell models. Ghatan et al. (2000) reported that p38 MAPK played a key role in NO-mediated neuronal death by stimulating the translocation of Bax to the mitochondria, which in turn triggered the apoptotic cascade. Neuronal apoptosis is one of the factors contributing to the poor prognosis of t-SCI (Niu and Yip, 2011). After t-SCI, some cells at the lesion site die because of post-traumatic necrosis, whereas others die by apoptosis (Byrnes et al., 2007). The apoptotic cascade can be activated in oligodendrocytes, microglia, and perhaps, astrocytes, and particularly in neurons (Liu et al., 1997; Beattie et al., 2000), which may be responsible for the serious dysfunction of patients with t-SCI (Mizuno et al., 1998; Mattson, 2000). Previous studies revealed that decreased apoptosis can promote functional improvement in t-SCI rats (Wang et al., 2017). DJ-1 exerts anti-apoptotic effects by reducing ROS. Additionally, DJ-1 can modulate the p38 MAPK pathway via direct physical interactions with its upstream protein, the ASK1, to reduce apoptosis (Mo et al., 2010).

In the present study, Western blotting indicated that DJ-1 was significantly increased after t-SCI induction. The ROS level, p-p38 MAPK/p38 MAPK ratio, and CC-3 level was significantly elevated, whereas the Bcl-2/Bax ratio was significantly reduced after t-SCI. Furthermore, treatment with DJ-1 siRNA significantly downregulated the protein level of DJ-1, elevated the ROS level, p-p38 MAPK/p38 MAPK ratio, and CC-3 level, and reduced the Bcl-2/Bax ratio. These results indicated that initiation of the ROS-induced apoptosis occurred after t-SCI, and DJ-1 showed preventative effects on ROS-induced apoptosis and functioned as a neuroprotective protein. Treatment with NaB significantly increased the expression level of DJ-1. Moreover, NaB treatment significantly decreased the ROS level, p-p38 MAPK/p38 MAPK ratio, and CC-3 level and elevated the Bcl-2/Bax ratio. DJ-1 siRNA significantly reversed these beneficial effects. TEM analysis also revealed abnormal subcellular structures and an increased proportion of mitochondrial vacuolization in t-SCI + vehicle group, indicating that neuronal apoptosis was induced by t-SCI. NaB treatment reversed t-SCI-induced apoptosis to some extent. The results confirmed that NaB treatment alleviated ROS-induced apoptosis by upregulating DJ-1 expression, which agrees with the results of previous studies and further supports the crucial anti-apoptotic effects of DJ-1 in t-SCI rats.

The ability to respond to oxidative stress is the best-established characteristic of DJ-1 (Canet-Aviles et al., 2004). Under oxidative stress, DJ-1 is transformed to a cysteine sulfinic acid (Cys106-SO_2_) via oxidation of its Cys106 residue as a post-translational modification (Blackinton et al., 2009b). It was reported that oxidation of Cys106 of DJ-1 contributed to its protective effects, while the absence of Cys106 oxidation led to the loss of DJ-1’s protective function (Blackinton et al., 2009b). Additionally, excessive oxDJ-1 enables cells to commit to apoptosis (Cao et al., 2014). In patients and animal models of PD, oxDJ-1 was increased in unmedicated PD, while drug therapy lowered oxDJ-1 levels, suggesting oxDJ-1 played an important role in PD and was a potential biomarker for PD (Saito, 2014; Saito et al., 2014; Mita et al., 2018; Yamagishi et al., 2018). Thus, we also tested the expression of oxDJ-1 and found that oxDJ-1 was significantly increased after t-SCI induction. DJ-1 siRNA, NaB, or NaB+DJ-1 siRNA significantly reduced the expression of oxDJ-1. Under t-SCI, DJ-1 can be oxidized into oxDJ-1, resulting in elevation of oxDJ-1 levels. DJ-1 siRNA reduced the levels of DJ-1 and oxDJ-1. DJ-1 upregulated by NaB can reduce ROS through self-oxidation (Taira et al., 2004) and form oxDJ-1, which was thought to cause upregulation oxDJ-1. However, we found a decrease in oxDJ-1 after NaB treatment. These results indicated that NaB treatment elevated the level of DJ-1 and eliminated ROS through other more powerful pathways, which reduced the oxidation of ROS toward DJ-1 and led to a decrease in oxDJ-1. In fact, self-oxidation is not the main antioxidant mechanism and contributes little to the antioxidant capacity of DJ-1 (Aleyasin et al., 2010).

Apart from neuronal apoptosis, t-SCI also induces edema and BSCB breakdown, which are the major pathological changes and contribute to a poor prognosis (Gao et al., 2018). In the clip compressive model of t-SCI, the compression forces applied to spinal cord can mutilate blood vessels, damage cellular components, and breakdown BSCB (Casella et al., 2006; Dulmovits and Herman, 2012). Sharma (2011) suggested that early spinal cord microvascular reactions after t-SCI led to spinal cord endothelial cell disturbances and caused the breakdown of BSCB functions. Destruction of the BSCB alters the microenvironment of the spinal cord (Wu et al., 2014) and thus causes the soakage of massive neutrophils and macrophages, contributing to severe inflammation, cell death, and durable neurological dysfunctions (Lee et al., 2012). In contrast, a reduction in BSCB breakdown results in significant neuroprotection (Wu et al., 2014). Spinal cord edema results from excessive SCWC both in the extracellular and intracellular spaces which can result from trauma, ischemia, and inflammation (Nesic et al., 2006). There are two main types of spinal cord edema: cytotoxic edema and vasogenic edema. T-SCI causes perivascular astrocyte endfeet swelling and dysfunction. As a result, excessive water enters the cells and leads to cytotoxic edema, further destroying the tight junction and vascular basal lamina, causing water to exudate from blood vessels and form vasogenic edema (Wang et al., 2011). Additionally, spinal cord edema can exacerbate destruction of the BSCB. In other words, spinal cord edema and BSCB destruction may exhibit reciprocal causation (Nesic et al., 2006). In our study, we found that t-SCI led to an increase in SCWC and EB extravasation, indicating edema and BSCB destruction of the injured spinal cord. However, these effects were significantly reversed by NaB treatment.

Overall, NaB treatment elevated the expression level of DJ-1, and then effectively reduced ROS-induced neuronal apoptosis. Additionally, NaB reduced spinal cord edema and BSCB destruction. At the macro level, the decreases in the BBB and IPT scores induced by t-SCI were significantly increased by NaB treatment at 21- and 28-days post-injury, indicating improvement in neurological functions. However, the macroscopic functional improvement results from a combination of multiple micro-events, which are not limited to the alleviation of neuronal apoptosis, spinal cord edema, or BSCB destruction. As a result, the improvement in neurological function is delayed in time with respect to improvement at the molecular and cellular levels as shown in our experiment. This indicates that other pathogenic factors contribute to the neurological impairment of t-SCI, which requires further analysis.

The DJ-1 gene belongs to the Thi/PfpI superfamily (Wang et al., 2018), which is located at chromosome 1p36 and encodes a ubiquitous protein consisting of 189 amino acids (Van Duijn et al., 2001). Among its biological functions, the most important is protecting against oxidative stress (Kahle et al., 2009). The main anti-oxidative stress mechanisms of DJ-1 include multiple aspects. First, DJ-1 is a redox protein; it removes ROS *in vitro* and *in vivo* by self-oxidation (Taira et al., 2004). The expression of DJ-1 is induced by oxidative stresses (Kinumi et al., 2004). In the three cysteine residues of DJ-1, Cys106 is the most sensitive amino acid toward intracellular oxidative stress. DJ-1 scavenges ROS when the Cys106 residue is oxidized to the acid subtype (Canet-Aviles et al., 2004). Second, DJ-1 shows molecular chaperone activity that is sensitive to redox reaction, helping cells resist harmful events induced by oxidative stress (Zhou et al., 2006). Third, DJ-1 acts as a transcriptional coactivator that can promote the transcription of glutathione and SOD (Zhong and Xu, 2008) and regulate the activity of peroxiredoxin-2, a significant antioxidant enzyme (Qu et al., 2007), which in turn decreases ROS levels. It has also been reported that nuclear factor erythroid 2-related factor-2, a transcription factor, can be stabilized by DJ-1, promoting its shift from the cytoplasm to the nucleus and then upregulating the expression of antioxidant genes (Malhotra et al., 2008). Fourth, DJ-1 interacts with several regulatory molecules in the nucleus to exert synergistic transcriptional regulatory actions (Sekito et al., 2006). Fifth, DJ-1 can modulate the function of mitochondria by maintaining the activity of mitochondrial complex 1, reducing ROS in the mitochondria, and preserving the mitochondrial membrane potential and mitochondrial shape (Krebiehl et al., 2010). The activity of mitochondrial complex 1 was downregulated in DJ-1 knockdown cells (Canet-Aviles et al., 2004; Ooe et al., 2005) and DJ-1 knockout mice (Goldberg et al., 2005; Kim et al., 2005b).

Moreover, previous studies showed that DJ-1 promoted the phosphorylation of Akt for activation, which in turn protected against oxidative stress damage. Akt is a serine/threonine protein kinase that participates in many aspects of cell activity including cell survival, growth, proliferation, differentiation, metabolism, and death (Manning and Toker, 2017). Akt is involved in diverse physical and pathological processes during CNS injuries (Ko et al., 2016). Additionally, Akt has many phosphorylation sites, among which the phosphorylation of Ser473 in the hydrophobic motif causes maximal activation (Manning and Toker, 2017). It was also reported that DJ-1 promoted the phosphorylation of Akt at Ser473 (Kim et al., 2005a; Aleyasin et al., 2010; Wang et al., 2014). As a result, we examined Ser473 phosphorylation of Akt in this study. Western blotting indicated that the expression level of DJ-1 was increased starting at 3 h after t-SCI, reached a peak at 24h, and then gradually decreased at 48 and 72 h. Interestingly, the p-Akt levels showed a similar trend. Additionally, the p-Akt level was decreased following knockdown of DJ-1, while it was increased following an increase in DJ-1 after NaB treatment. However, some studies showed that DJ-1 promoted the phosphorylation of Akt at other sites, such as Ser505 (Yang et al., 2005) and Thr308 (Gorner et al., 2007; Zhang et al., 2016), which were also involved in preventing oxidative stress. Whether phosphorylation at these sites is important remains unclear and requires further analysis.

Activated Akt can help multiple types of cells to resist ROS-induced injuries via diverse pathways, as well as by modulating the expression of antioxidant enzymes. The major antioxidant enzymes include glutathione peroxidase, catalase, and particularly the SOD family, which help to degrade ROS to reduce damage to DNA, proteins, lipids, and other cellular components (Davis and Pennypacker, 2017). The SOD family has three isoforms, SOD1, SOD2, and SOD3, among which SOD2 is ubiquitously expressed and one of the major antioxidant enzymes responsible for scavenging ROS in the mitochondria (Chan, 2005). Previous studies reported that decreased SOD2 activity was associated with AD and PD (Wiener et al., 2007; Belluzzi et al., 2012). SOD2 knockout mice were more sensitive to oxidative stress after cerebral ischemia (Kim et al., 2002; Mehta et al., 2011), while increased SOD2 alleviated ischemic brain injury (Davis and Pennypacker, 2017). Akt activation promoted the expression of SOD2 to exert cell protective effects (Saha et al., 2016). As a result, we detected SOD2 in our study and found that SOD2 expression was consistent with p-Akt expression.

These results suggested that upregulation of DJ-1 promoted the expression of SOD2 by activating Akt, which in turn disintegrated ROS in rats with t-SCI. As a result, ROS-induced apoptosis was also reduced. We further utilized the highly selective Akt inhibitor MK2206 to confirm these results. MK2206 effectively inhibits the phosphorylation of Akt, and thus prevents the activation of downstream molecules. Western blotting showed that MK2206 treatment significantly blocked the anti-oxidative stress effects of DJ-1. Double IF staining showed that the proportions of CC-3- and TUNEL-positive neurons were increased significantly post-injury, indicating neuronal apoptosis. Treatment with NaB significantly reduced neuronal apoptosis; however, this decrease was reversed by MK2206. Notably, given that MK2206 alone did not obviously alter neuronal apoptosis, confirming mediation of the protective effects of DJ-1 via phosphorylation of Akt. The reason for this may be that MK2206 could not inhibit Akt under injury without additional interventions.

**FIGURE 12:**
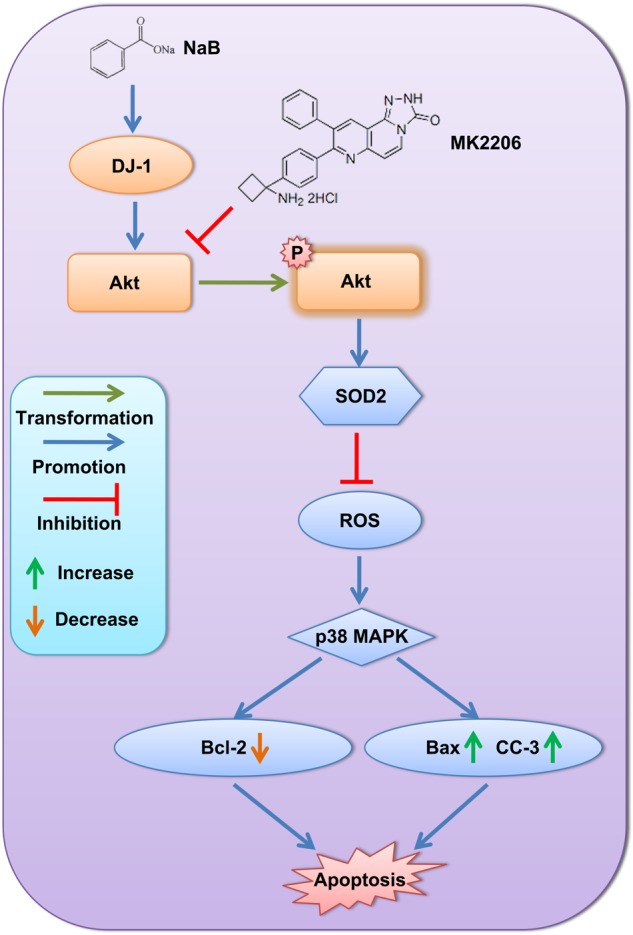
Schematic map of the potential signaling pathway showing alleviation of neuronal apoptosis by NaB via DJ-1/Akt-related anti-oxidative stress pathway.

The results of our study suggested that NaB reduced ROS and ROS-induced neuronal apoptosis by upregulating DJ-1 and its downstream proteins, p-Akt and SOD2. A schematic map of the potential signaling pathway is shown in Figure 12.

NaB is a metabolite of cinnamon and FDA-approved food additive, which has wide applications in the food industry. It is non-toxic and can be administered as a solution in drinking water. Long-term intake of a 2% NaB aqueous solution is safe and shows no side effects in experimental mice (Toth, 1984). A minor amount of NaB is expelled in the urine of humans after intake (Kubota and Ishizaki, 1991). The typical FDA-approved dose of NaB for patients with hyperammonemia is 4–10 g/day, which was further increased during treatment of acute stage hyperammonemia (Sushma et al., 1992; Misel et al., 2013). As a potential drug for treating CNS disorders, NaB shows obvious advantages. First, NaB can be administered orally, which is convenient and results in good compliance; second, NaB is a liposoluble molecule capable of diffusing through the BSCB. NaB shows considerable accumulation in the cerebrospinal fluid, making it an effective agent for treating CNS disorders (Khasnavis and Pahan, 2012). However, the optimal NaB dosage and duration of treatment of CNS injury, particularly for t-SCI requires further analysis.

## Limitations

There were some limitations to our study. First, DJ-1 can exert neuroprotective effects through a variety of pathways; such pathways are often linked with each other, whereas we only studied the anti-oxidative stress and anti-ROS-induced apoptosis effects. Further studies are needed to evaluate other mechanisms. Second, in our study, NaB treatment alleviated spinal cord edema and BSCB destruction; however, its exact molecular mechanism and the mechanism of action of DJ-1 should be further examined. Third, in addition to neurons, large numbers of white matter fibers exist in the spinal cord. As a result, white matter injury is also a critical pathological process during t-SCI. Whether DJ-1 is involved in preventing white matter injury should be further evaluated.

## Conclusion

This is the first study to demonstrate the neuroprotective role of NaB in a rat model of t-SCI. NaB upregulated DJ-1, and thus reduced ROS and ROS-induced neuronal apoptosis via Akt phosphorylation, and increased SOD2 expression. Given the crucial involvement of ROS in t-SCI, DJ-1 may be an important target while NaB is a promising therapeutic agent for treating t-SCI.

## Ethics Statement

This study was carried out in accordance with the recommendations of the NIH guidelines. The protocol was approved by the ethics committee of Zhejiang University.

## Author Contributions

BQ, JZ, TL, and GY performed the analysis of the t-SCI model. FY, WX, ZZ, and BQ performed the Western blots. TZ and JZ prepared the figures. LG and GY performed the IF staining. TL and JL performed the data analysis. LG, YZ, and GC designed the experiments. LG, ZZ, and WX contributed to the writing and editing of the manuscript. YZ and GC reviewed and revised the manuscript.

## Conflict of Interest Statement

The authors declare that the research was conducted in the absence of any commercial or financial relationships that could be construed as a potential conflict of interest.

## Abbreviations

AD: Alzheimer’s disease
ANOVA: analysis of variance
BBB: Basso, Beattie, and Bresnahan
BSCB: blood-spinal cord barrier
CC-3: cleaved caspase-3
CNS: central nervous system
EB: Evans Blue
IF: immunofluorescence
IPT: inclined plane test
i.t.: intrathecal injection
MS: multiple sclerosis
NaB: natrium benzoate
oxDJ-1: oxidized DJ-1
PD: Parkinson’s disease
ROS: reactive oxygen species
SCWC: spinal cord water content
SD: Sprague-Dawley
siRNA: small interfering RNA
SOD: superoxide dismutase
TEM: transmission electron microscopy
t-SCI: traumatic spinal cord injury
TUNEL: terminal deoxynucleotidyl transferase-mediated dUTP nick end labeling
